# A Remote Control Strategy for an Autonomous Vehicle with Slow Sensor Using Kalman Filtering and Dual-Rate Control

**DOI:** 10.3390/s19132983

**Published:** 2019-07-06

**Authors:** Ángel Cuenca, Wei Zhan, Julián Salt, José Alcaina, Chen Tang, Masayoshi Tomizuka

**Affiliations:** 1 Instituto Universitario de Automática e Informática Industrial, Universitat Politècnica de València, 46022 València, Spain; 2 Mechanical Engineering Department, University of California, Berkeley, CA 94720, USA

**Keywords:** autonomous vehicle, slow sensor, Kalman filter, networked control, dual-rate control

## Abstract

This work presents a novel remote control solution for an Autonomous Vehicle (AV), where the system structure is split into two sides. Both sides are assumed to be synchronized and linked through a communication network, which introduces time-varying delays and packet disorder. An Extended Kalman Filter (EKF) is used to cope with the non-linearities that appear in the global model of the AV. The EKF fuses the data provided by the sensing devices of the AV in order to estimate the AV state, reducing the noise effect. Additionally, the EKF includes an *h*-step-ahead state prediction stage, which, together with the consideration of a packet-based control strategy, enables facing the network-induced delays. Since the AV position is provided by a camera, which is a slow sensing device, a dual-rate controller is required to achieve certain desired (nominal) dynamic control performance. The use of a dual-rate control framework additionally enables saving network bandwidth and deals with packet disorder. As the path-tracking control algorithm, pure pursuit is used. Application results show that, despite existing communication problems and slow-rate measurements, the AV is able to track the desired path, keeping the nominal control performance.

## 1. Introduction

In this work, a remote control solution is proposed for Autonomous Vehicle (AV) path tracking. The solution fits in the field of Networked Control Systems (NCSs) [1,2,3,4], which is a prolific control area that addresses control scenarios where different devices share a common communication link. The use of a shared communication network introduces some advantages like cost reduction, reconfigurability, and ease of installation and maintenance, but also some drawbacks such as the possible occurrence of network-induced delays [5,6,7,8,9,10], packet dropouts [11,12,13], packet disorder [14,15,16], and network bandwidth constraints [17,18,19]. In this work, the drawbacks considered in the network are time-varying delays and packet disorder.

An AV can be defined as a vehicle that is capable of intelligent motion and action without human input [20]. AVs have attracted the attention of the scientific community due to the large number of applications in which they can be involved (for instance, target tracking [21], surveillance [22], transportation [23], self-driving cars [24], etc.). In our work, a target tracking application is developed, where the AV concretely is a differential robot. As the path tracking algorithm, pure pursuit [25,26,27] has been chosen. The hierarchical structure of the AV (see, e.g., [28]) is split into two sides, with the communication network between them. In particular, the motion planning and vehicle control layers are located on-board, whereas upper levels of the structure such as decision-making, path generation, and monitoring are located at the remote server. In this way, safety-critical tasks are executed on-board in real-time, whereas longer term computations, which mostly affect performance and are generally not real-time critical, are executed in the remote server.

The present work is mainly focused on designing a control solution at the local side, which will integrate state estimation, packet-based control, and dual-rate control. The main idea is to generate a set of path references from way points at the remote server, and send them to the AV through the network. Then, the control level is able to compute a delay-free control signal to be applied to the AV and generate a set of future state estimations, which is sent to the remote server for upper level purposes such as displaying variables, making decisions (e.g., trajectory replanning), etc.

As is well known, AV path tracking involves non-linearities [29]. Sensed variables must be accurately estimated and corrected in order to provide the motion planning and control layers with reliable data. For this purpose, different filtering approaches can be used, such as the Kalman filter (see, for example, [30,31,32]) and the $H_{\infty }$ filter (see, for instance, [32,33]), among others. The attractiveness of the Kalman filter lies in the fact that it is the one estimator that results in the smallest possible standard deviation of the estimator error. However, when the goal is to minimize the worst-case estimation error, the $H_{\infty }$ filter is a better option. The $H_{\infty }$ filter provides a rigorous method for dealing with systems that have model uncertainty, but is more sensitive to the design parameters (weighting functions) than the Kalman filter. As a conclusion, $H_{\infty }$ theory shows us the optimal way to robustify the Kalman filter, this theory being more abstract and complicated than the one underlying the Kalman filtering.

Focusing on the Kalman filter, it is widely used in its extended and unscented versions (see, e.g., [29,30,31,34]). In this work, the Extended Kalman Filter (EKF) has been chosen not only to estimate the non-linear behavior of the AV, reducing the process and measurement noise effect, but also to fuse all the data provided by the different sensors in order to be used by the control algorithm. The sensing devices used in our path tracking application are two encoders, an Inertial Measurement Unit (IMU) and a zenithal camera, which respectively provide the angular velocities of each wheel, the AV orientation, and the AV position. In our proposal, the EKF is complemented with an *h*-step-ahead state prediction stage, which, together with the integration of packet-based control strategies in the control solution, enables compensating for time-varying network-induced delays.

Packet-based control [35,36] is usually employed to decrease the communication rate by simultaneously sending a set of data in each transmission. In our work, the main aim of including this technique in the control solution is two-fold:

1. To provide the motion planning and control layers with a set of *h*-step-ahead path references. From this set and after successively iterating the different components of these layers in the current sensing period, a set of *h*-step-ahead control action estimates can be computed by following a delay-free control algorithm.
2. To supply the upper layers of the AV structure included at the remote server with a set of *h*-step-ahead state predictions (computed by the motion planning and control layers in every sensing period), which is updated irrespective of the delay when a new packet is received.

Then, for implementation purposes, the time-varying network-induced delay is not required to be measured and compensated for. This is a relevant aspect of this work, since it makes the solution applicable to a wide range of wireless control applications where the time delay is difficult to measure. This working mode is possible because an accurate system model and an acceptable level of noise are assumed, and hence, the difference between actual and estimated signals should be negligible. In addition, the possible divergences between both signals can be corrected by the EKF.

In dual-rate control [37,38,39,40], a slower sensing rate in comparison to a faster actuation can be assumed. As mentioned, in this work, a zenithal camera is used to sense the AV position, which is provided at a slow rate due to technical constraints. Despite sensing in this way, the fast actuation enables achieving acceptable control properties. In addition, dual-rate control techniques provide two-fold benefits: (i) to reduce the amount of transmissions through the network, which results in bandwidth saving; (ii) to elude packet disorder, selecting the sampling period to be larger than the largest network-induced time delay. As a statistical distribution for the delay is assumed to be known [41], the largest delay can be easily found. In the present work, due to the broad knowledge of PID controllers in academic and industrial environments, a dual-rate PID control scheme is considered.

In summary, the main contribution of the present work is the development of a novel and complete remote control approach for an AV, where an EKF (including an *h*-step-ahead state prediction stage), packet-based control, the pure pursuit path tracking algorithm, and a dual-rate dynamic controller are systematically brought together in order to keep the nominal control properties when path tracking and considerably lessen resource usage such as network bandwidth. These facts are reached despite existing wireless communication problems such as time-varying delays and packet disorder, and the appearing process and measurement noise signals. The nominal control performance is defined by means of an ideal fast-rate control framework, where no noise and delay are considered.

In Section 2, the novel control structure is presented. In addition, some details about how the network-induced delays can be faced are posed. In Section 3, the non-linear model of the AV is introduced, and then, the control design of the motion planning and control layers of the AV structure is formulated. Section 4 presents some cost indexes to measure and compare control performance among the proposed control solution and other approaches. Section 5 shows the main benefits of the control proposal by means of a Truetime application [42]. Finally, Section 6 summarizes the main conclusions of the work.

## 2. Problem Scenario

**Figure 1:**
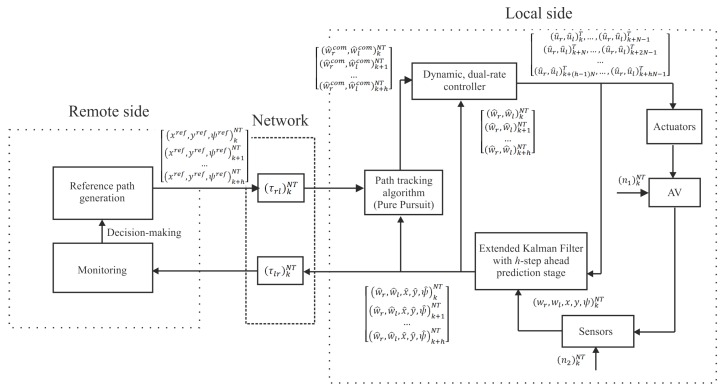
Remote control structure for an autonomous vehicle.

The overall control scheme is depicted in Figure 1. The proposed structure includes two sides: (i) the local side, where the AV is located together with different actuators and sensors (which can introduce measurement noise) and where motion planning and vehicle control layers are implemented; and (ii) the remote side, where upper level layers of the hierarchical structure of the AV are included, in such a way that the remote server is in charge of generating the path reference based on way points, displaying and monitoring system data, and making decisions from this information. A wireless network is used to connect both sides, which introduces some communication problems such as time-varying delays and packet disorder. In the next subsections, these problems are formally described, and the working mode of the control structure is presented.

### 2.1. Control Structure

There are three main keys to the control solution:

1. Fusing all the data provided by the different sensors (encoders, IMU, camera) by means of an Extended Kalman Filter (EKF) in order to estimate the state of the AV, reducing the noise effect. The extended version of the Kalman Filter is needed due to the non-linear nature of the AV.
2. Including an *h*-step-ahead state prediction stage in the EKF, using a packet-based control strategy, for the purpose of dealing with network-induced delays, and providing the remote side with future, estimated data.
3. Integrating dual-rate control with a view toward achieving the desired (nominal) control specifications, coping with slow sensing and packet disorder.

The control structure uses two different periods: *T* as the actuation period and NT as the sensing period, $N\in N^{+}$ being the multiplicity between the two periods of the dual-rate control scheme [38]. Let us respectively denote ${\left(.\right)}_{k}^{T}$ and ${\left(.\right)}_{k}^{NT}$ as a *T*-period and an NT-period signal or variable, where k∈N are iterations in the corresponding period. Communication between the two network sides is carried out in the sensing period NT, i.e., at instants kNT, which helps save network bandwidth; concretely, the use of the network is *N*-times lower than that produced by a conventional single-rate control in period *T*.

In more detail, the control structure works as follows:

- At the current instant *kNT*, the remote side generates a set of *h* path references, which includes from the reference to be used at instant *k*, i.e., ${\left(x^{ref},y^{ref},\psi^{ref}\right)}_{k}^{NT}$, to the reference to be used at instant (k+h)NT, i.e., ${\left(x^{ref},y^{ref},\psi^{ref}\right)}_{k+h}^{NT}$. The set is composed of reference positions $\left(x^{ref},y^{ref}\right)$ and reference yaw angle $\psi^{ref}$, and it is sent to the local side in a packet.
- The local side gets the current system state ${\left(w_{r},w_{l},x,y,\psi \right)}_{k}^{NT}$, which coincides with the system output in this work, being affected by the process and measurement noises, ${\left(n_{1}\right)}_{k}^{NT}$ and ${\left(n_{2}\right)}_{k}^{NT}$, respectively. The state is composed of angular velocities $\left(w_{r},w_{l}\right)$, positions (x,y), and yaw angle ψ.
- The next estimation of the system state ${\left(\hat{w_{r}},\hat{w_{l}},\hat{x},\hat{y},\hat{\psi }\right)}_{k}^{NT}$ is computed via an Extended Kalman Filter (EKF). This estimation carried out by the EKF is actually the correction of the state.
- From this state and the path reference for the instant kNT, i.e., ${\left(x^{ref},y^{ref},\psi^{ref}\right)}_{k}^{NT}$, received in the previous packet after the remote-to-local delay ${\left(\tau_{rl}\right)}_{k}^{NT}$, the path tracking algorithm (pure pursuit in this case) computes the dynamic reference, or command, for the instant kNT, i.e., ${\left({\hat{w}}_{r}^{com},{\hat{w}}_{l}^{com}\right)}_{k}^{NT}$.
- From this dynamic reference and the estimated angular velocities ${\left({\hat{w}}_{r},{\hat{w}}_{l}\right)}_{k}^{NT}$, the dynamic, dual-rate controller computes the control signal to be applied to the AV, i.e., $\left\{{\left({\hat{u}}_{r},{\hat{u}}_{l}\right)}_{k}^{T},\ldots ,{\left({\hat{u}}_{r},{\hat{u}}_{l}\right)}_{k+N-1}^{T}\right\}$, which are the control actions in period *T* for the right and left motors, respectively, inside the sensing period kNT. As a uniform actuation pattern, the actuation occurs at uniformly-spaced instants kNT+lT (l=0,1,…,N−1) under Zero Order Hold (ZOH) conditions inside the sensing period. That is, ${\left({\hat{u}}_{r},{\hat{u}}_{l}\right)}_{k}^{T}$ is applied at kNT, ${\left({\hat{u}}_{r},{\hat{u}}_{l}\right)}_{k+1}^{T}$ is injected at kNT+T, and so on, up to ${\left({\hat{u}}_{r},{\hat{u}}_{l}\right)}_{k+N-1}^{T}$, which is actuated at kNT+(N−1)T.
- From this control signal and the estimated state for the instant kNT, the *h*-step-ahead state prediction stage is able to compute the estimation of the state for the instant (k+1)NT, i.e., ${\left({\hat{w}}_{r},{\hat{w}}_{l},\hat{x},\hat{y},\hat{\psi }\right)}_{k+1}^{NT}$. This computation is carried out in an open loop. Iterating the control loop *h* times, the local side can obtain the *h* state estimations to be sent in a packet to the remote side. Therefore, when the remote server receives the packet at the current time instant *kNT* and after the local-to-remote delay ${\left(\tau_{lr}\right)}_{k}^{NT}$, it can manage future system information, for example, to be displayed and to make decisions.

### 2.2. Time-Varying Delays, and Packet Disorder

Assuming synchronization between both network sides [43], time-varying delays can be measured for both communication links. From off-line experiences on the communication network, a statistical distribution of the delay can be obtained and, hence, the maximum delay $\tau_{max}$. Packet disorder may appear when $NT<\tau_{max}$. In the present work, by taking advantage of the use of dual-rate control to deal with slow sensing, a straightforward solution is considered to avoid packet disorder, that is setting up the sampling period NT to be larger than the maximum time delay $\tau_{max}$, i.e., $NT>\tau_{max}$.

**Figure 2:**
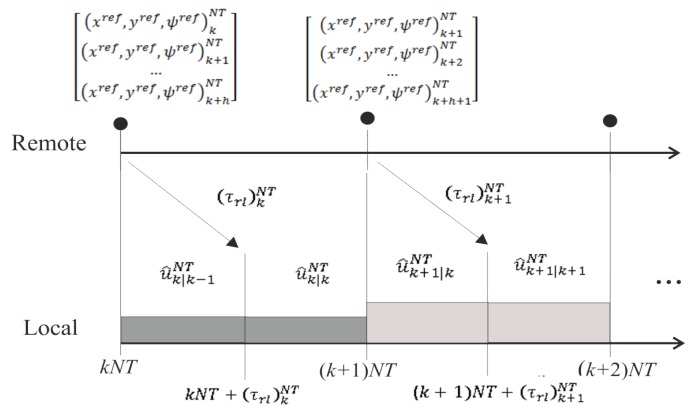
Time axis example for the remote-to-local channel.

**Figure 3:**
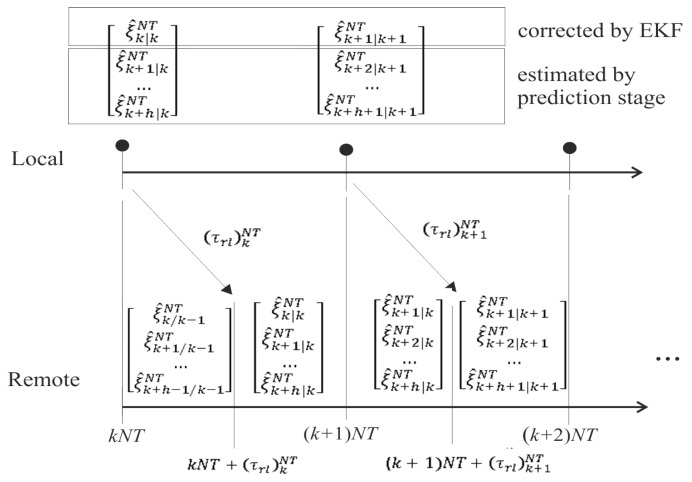
Time axis example for the local-to-remote channel.

In order to cope with time-varying delays, the control solution integrates a packet-based strategy and predictor-based control. Next, details about how the delays can be faced are given:

- Remote-to-local delays ${\left(\tau_{rl}\right)}_{k}^{NT}$: By implementing the packet-based strategy, the packet with the set of references generated by the remote server from the instant kNT to the instant (k+h)NT arrives after the delay ${\left(\tau_{rl}\right)}_{k}^{NT}$ at the local side. As a consequence of integrating predictor-based control and assuming an accurate system model and an acceptable level of noise, this delay will not affect the control system. The reason is that, since the reference for the current instant was received in the previous delivery, the consequent estimated control action is already being injected from the beginning of the current period. When the packet is received, a new control action is computed from the corrected state. This control action will be very similar to the estimated one, and hence, an insignificant change is produced in the control signal. Note that possible changes in the reference due to decision-making tasks are recommended to be included at least from instant (k+1)NT of the set of references in order to keep the described working mode, avoiding the delay effect. Figure 2 depicts a time axis example of this communication channel, where, for the sake of simplicity, a single-rate control at NT is considered. Notation ${\hat{u}}_{k+1|k}^{NT}$ means the estimated control action to be applied at instant (k+1)NT, which is calculated at instant *kNT* from the reference ${\left(x^{ref},y^{ref},\psi^{ref}\right)}_{k+1}^{NT}$. As shown, when the packet is received, a new control action is computed, which is practically the same as that previously calculated from the preceding delivery, i.e., ${\hat{u}}_{k/k-1}^{NT}≅{\hat{u}}_{k/k}^{NT}$ and ${\hat{u}}_{k+1/k}^{NT}≅{\hat{u}}_{k+1/k+1}^{NT}$.
- Local-to-remote delays ${\left(\tau_{lr}\right)}_{k}^{NT}$: Similar to the other link, by implementing the packet-based strategy, the packet with the set of estimations generated by the EKF and the *h*-step-ahead state prediction stage from instant kNT to instant (k+h)NT arrives after the delay ${\left(\tau_{lr}\right)}_{k}^{NT}$ at the remote side. The working mode is as follows: from the beginning of the period, a set of state estimates is used, and when a new packet arrives, this set is replaced with the received one, which includes state correction. Figure 3 depicts a time axis example of this communication channel. The notation ${\hat{\xi }}_{k+1|k}^{NT}$ means the state estimated for the instant (k+1)NT at the instant kNT, i.e., ${\left(\hat{w_{r}},\hat{w_{l}},\hat{x},\hat{y},\hat{\psi }\right)}_{k+1|k}^{NT}$. The estimation ${\hat{\xi }}_{k|k}^{NT}$ is actually the state corrected by the EKF, and hence, the state estimate ${\hat{\xi }}_{k|k-1}^{NT}$ can be replaced with its correction ${\hat{\xi }}_{k|k}^{NT}$. From this correction, the *h*-step-ahead state prediction stage estimates the rest of the state values $\left\{{\hat{\xi }}_{k+1|k}^{NT},\ldots ,{\hat{\xi }}_{k+h-1|k}^{NT}\right\}$ in order to replace the previous estimations $\left\{{\hat{\xi }}_{k+1|k-1}^{NT},\ldots ,{\hat{\xi }}_{k+h-1|k-1}^{NT}\right\}$. Additionally, the prediction stage generates a new state estimate ${\hat{\xi }}_{k+h|k}^{NT}$ to complete the set of predicted states. Excluding this new value and considering an accurate model and an acceptable level of noise, the difference between the previous and the current set of state values should be negligible.

## 3. Motion Planning and Control Solution Design

In the following subsections, each component of the motion planning and control layers of the AV structure will be defined in detail. The systematic combination of these components (EKF including *h*-step-ahead state prediction stage, pure pursuit path tracking algorithm, and dual-rate dynamic controller) is the most relevant contribution of this work. It enables maintaining the nominal control performance when path tracking and reduces the network bandwidth usage, despite the existence of time-varying network-induced delays and packet disorder and the appearance of process and measurement noise signals. Next, first of all, the AV model will be presented.

### 3.1. Plant Modeling

In this work, a non-linear kinematic model was utilized by the EKF in order to fulfill state estimation. Additionally, a dynamic relation between the control signal and the rotational velocity of the wheel was needed in order to design the dual-rate dynamic controller.

#### 3.1.1. Kinematic Model

The kinematic model represents the AV velocity evolution in a fixed inertial frame. From the AV linear and rotational velocities in sampling period NT, i.e., $v_{k}^{NT}$ and $\omega_{k}^{NT}$, respectively, the AV position and orientation in the time period NT, i.e., ${\left(x,y,\psi \right)}_{k}^{NT}$, can be deduced as follows [44]:

(1) $${\left(\begin{array}{c} x\\ y\\ \psi \end{array}\right)}_{k}^{NT}={\left(\begin{array}{c} x\\ y\\ \psi \end{array}\right)}_{k-1}^{NT}+\left(\begin{array}{c} v_{k}^{NT}NT\cos\left(\psi_{k-1}^{NT}+\omega_{k}^{NT}NT\right)\\ v_{k}^{NT}NT\sin\left(\psi_{k-1}^{NT}+\omega_{k}^{NT}NT\right)\\ \omega_{k}^{NT}NT \end{array}\right)$$

$\mathrm{for}\;k\in N_{\geq 1}$, where ${\left(x,y,\psi \right)}_{0}^{NT}$ is the initial position and orientation and where the AV linear and rotational velocities come from:

(2) $${\left(\begin{array}{c} v\\ w \end{array}\right)}_{k}^{NT}=\left(\begin{array}{cc} 1/2 & 1/2\\ 1/2b & -1/2b \end{array}\right){\left(\begin{array}{c} v_{r}\\ v_{l} \end{array}\right)}_{k}^{NT}$$

being *b* half of the distance between the wheels and ${\left(v_{r},v_{l}\right)}_{k}^{NT}$ the linear velocities for each wheel, which are defined from the radius of the wheels, $r_{r}$ and $r_{l}$, and the consequent rotational velocities sensed in period NT, ${\left(w_{r},w_{l}\right)}_{k}^{NT}$, as follows:

(3) $${\left(\begin{array}{c} v_{r}\\ v_{l} \end{array}\right)}_{k}^{NT}=\left(\begin{array}{cc} r_{r} & 0\\ 0 & r_{l} \end{array}\right){\left(\begin{array}{c} w_{r}\\ w_{l} \end{array}\right)}_{k}^{NT}$$

#### 3.1.2. Dynamic Model

The dynamic model represents the relation between the control signal and the rotational velocity for each wheel. Considering state-space representation, the model in sampling period *T* takes this form:

(4) $$\begin{array}{c} \left\{\begin{array}{cc} {\left(x_{p}\right)}_{k+1}^{T} & =A\cdot {\left(x_{p}\right)}_{k}^{T}+B\cdot {\hat{u}}_{k}^{T}+{\left(n_{1}\right)}_{k}^{T}\\ {\left(y_{p}\right)}_{k}^{T} & =C\cdot {\left(x_{p}\right)}_{k}^{T}+{\left(n_{2}\right)}_{k}^{T} \end{array}\right. \end{array}$$

where, for the sake of simplicity, let us name:

- ${\left(y_{p}\right)}_{k}^{T}$ as the output, that is the rotational velocity either for the right motor ${\left(w_{r}\right)}_{k}^{T}$ or for the left motor ${\left(w_{l}\right)}_{k}^{T}$ and
- ${\hat{u}}_{k}^{T}$ as the input, that is the control signal, regardless of the motor, ${\left(\hat{u_{r}}\right)}_{k}^{T}$ or ${\left(\hat{u_{l}}\right)}_{k}^{T}$.

In addition, ${\left(n_{1}\right)}_{k}^{T}$ is the process noise, ${\left(n_{2}\right)}_{k}^{T}$ the measurement noise, ${\left(x_{p}\right)}_{k}^{T}$ the process state (regardless of the motor), and A,B,C matrices with suitable dimensions. No unknown input is assumed.

Using Z-transform in period *T*, the input-output plant model for the control signal and the rotational velocity is represented as a discrete-time transfer function:

(5) $$G_{p}^{T}\left(z\right)=Y_{p}^{T}\left(z\right)/{\hat{U}}^{T}\left(z\right)$$

*z* being the *T*-unit operator.

The plant model in (4) admits a lifted representation [45] such as:

(6) $$\begin{array}{c} \left\{\begin{array}{cc} {\left(x_{p}\right)}_{k+N}^{T} & =A^{N}\cdot {\left(x_{p}\right)}_{k}^{T}+\sum_{c=0}^{N-1}A^{N-1-c}\cdot B\cdot {\hat{u}}_{k+c}^{T}+{\left(n_{1}\right)}_{k}^{T}\\ {\left(y_{p}\right)}_{k}^{T} & =C\cdot {\left(x_{p}\right)}_{k}^{T}+{\left(n_{2}\right)}_{k}^{T} \end{array}\right. \end{array}$$

which can be equivalently seen as a dual-rate sampled-data system, where the output in period NT, ${\left(y_{p}\right)}_{k}^{NT}$, i.e., either ${\left(w_{r}\right)}_{k}^{NT}$ or ${\left(w_{l}\right)}_{k}^{NT}$, is obtained from a sequence of inputs in period *T* included in the sensor period NT, i.e., ${\hat{U}}_{k}^{NT}={\left({\hat{u}}_{k}^{T},{\hat{u}}_{k+1}^{T},\ldots ,{\hat{u}}_{k+N-1}^{T}\right)}^{⊤}$, where ${\left(\cdot \right)}^{⊤}$ means the transpose function, such as:

(7) $$\begin{array}{c} \left\{\begin{array}{cc} {\left(x_{p}\right)}_{k+1}^{NT} & =A^{N}\cdot {\left(x_{p}\right)}_{k}^{NT}+\left(\begin{array}{cccc} A^{N-1}\cdot B & A^{N-2}\cdot B & \ldots & B \end{array}\right)\left(\begin{array}{c} {\hat{u}}_{k}^{T}\\ {\hat{u}}_{k+1}^{T}\\ \ldots \\ {\hat{u}}_{k+N-1}^{T} \end{array}\right)+{\left(n_{1}\right)}_{k}^{NT}\\ {\left(y_{p}\right)}_{k}^{NT} & =C\cdot {\left(x_{p}\right)}_{k}^{NT}+{\left(n_{2}\right)}_{k}^{NT} \end{array}\right. \end{array}$$

From (1)–(3) and (7), the global dynamic model for the AV was obtained. This model was used by the EKF to estimate the AV position and orientation.

### 3.2. Extended Kalman Filter, Including an *h*-Step-Ahead State Prediction Stage

Since the AV model is non-linear, the use of Kalman filtering for state estimation may be considered through a linearization procedure, which is based on the use of a matrix of partial derivatives, that is a Jacobian matrix. At each time step, the Jacobian matrix is evaluated with current predicted states. This procedure results in an EKF (see, e.g., [30,31,32]).

Let us denote the global non-linear dynamic model previously introduced by means of (1)–(3) and (7) as the next state-space representation:

(8) $$\begin{array}{c} \left\{\begin{array}{c} \xi_{k}^{NT}=f\left(\xi_{k-1}^{NT},{\left(n_{1}\right)}_{k-1}^{NT},{\hat{U}}_{k-1}^{NT}\right)\\ z_{k}^{NT}=h\left(\xi_{k}^{NT},{\left(n_{2}\right)}_{k}^{NT}\right) \end{array}\right. \end{array}$$

where we use the AV state $\xi_{k}^{NT}$ as ${\left({\left(w_{r},w_{l},x,y,\psi \right)}_{k}^{NT}\right)}^{⊤}$. As previously defined, ${\hat{U}}_{k}^{NT}$ is the control signal, and ${\left(n_{1}\right)}_{k}^{NT}$ and ${\left(n_{2}\right)}_{k}^{NT}$ are the process and measurement noises, respectively, which were both assumed to be zero mean multivariate Gaussian noises with covariance $Q_{k}^{NT}$ and $R_{k}^{NT}$, respectively.

Taking into account the notation introduced in Section 2.1, where ${\hat{\xi }}_{j|i}^{NT}$ means the state estimated for the instant jNT at the instant iNT, the prediction and correction steps of the EKF are defined as follows:

- Prediction of the next state ${\hat{\xi }}_{k|k-1}^{NT}$ and propagation of the covariance $P_{k|k-1}^{NT}$:
  (9) $$\begin{array}{cc} {\hat{\xi }}_{k|k-1}^{NT} & =f\left({\hat{\xi }}_{k-1|k-1}^{NT},{\left(n_{1}\right)}_{k-1}^{NT},{\hat{U}}_{k-1}^{NT}\right)\\ P_{k|k-1}^{NT} & =A_{k}^{NT}P_{k-1|k-1}^{NT}{\left(A_{k}^{NT}\right)}^{⊤}+L_{k}^{NT}Q_{k-1}^{NT}{\left(L_{k}^{NT}\right)}^{⊤} \end{array}$$
  $\mathrm{for}\;k\in N_{\geq 1}$, where ${\hat{\xi }}_{0}^{NT}=E\left[\xi_{0}^{NT}\right]$ and $P_{0}^{NT}=E\left[\left(\xi_{0}^{NT}-E\left[\xi_{0}^{NT}\right]\right){\left(\xi_{0}^{NT}-E\left[\xi_{0}^{NT}\right]\right)}^{⊤}\right]$, E[·] being the expectation, and where $A_{k}^{NT}$ and $L_{k}^{NT}$ are Jacobian matrices computed in order to linearize respectively the process model about the current state and about the process noise:
  (10) $$\begin{array}{cc} A_{k}^{NT} & ={\left.\frac{\partial f}{\partial \xi }\right|}_{{\hat{\xi }}_{k-1|k-1}^{NT},{\left(n_{1}\right)}_{k-1}^{NT},{\hat{U}}_{k-1}^{NT}}\\ L_{k}^{NT} & ={\left.\frac{\partial f}{\partial n_{1}}\right|}_{{\hat{\xi }}_{k-1|k-1}^{NT},{\left(n_{1}\right)}_{k-1}^{NT},{\hat{U}}_{k-1}^{NT}} \end{array}$$
- Prediction of the future output ${\hat{z}}_{k}^{NT}$ and computation of the Kalman filter gain $K_{k}^{NT}$:
  (11) $$\begin{array}{cc} {\hat{z}}_{k}^{NT} & =h\left({\hat{\xi }}_{k|k-1}^{NT},{\left(n_{2}\right)}_{k}^{NT}\right)\\ K_{k}^{NT} & =P_{k|k-1}^{NT}{\left(H_{k}^{NT}\right)}^{⊤}{\left(H_{k}^{NT}P_{k|k-1}^{NT}{\left(H_{k}^{NT}\right)}^{⊤}+M_{k}^{NT}R_{k}^{NT}{\left(M_{k}^{NT}\right)}^{⊤}\right)}^{-1} \end{array}$$
  where $H_{k}^{NT}$ and $M_{k}^{NT}$ are Jacobian matrices calculated in order to linearize respectively the output model about the predicted next state and about the measurement noise:
  (12) $$\begin{array}{cc} H_{k}^{NT} & ={\left.\frac{\partial h}{\partial \xi }\right|}_{{\hat{\xi }}_{k|k-1}^{NT},{\left(n_{2}\right)}_{k}^{NT}}\\ M_{k}^{NT} & ={\left.\frac{\partial h}{\partial n_{2}}\right|}_{{\hat{\xi }}_{k|k-1}^{NT},{\left(n_{2}\right)}_{k}^{NT}} \end{array}$$
- Correction of the state ${\hat{\xi }}_{k|k}^{NT}$ and correction of the covariance $P_{k|k}^{NT}$:
  (13) $$\begin{array}{cc} {\hat{\xi }}_{k|k}^{NT} & ={\hat{\xi }}_{k|k-1}^{NT}+K_{k}^{NT}\left(z_{k}^{NT}-{\hat{z}}_{k}^{NT}\right)\\ P_{k|k}^{NT} & =K_{k}^{NT}R_{k}^{NT}{\left(K_{k}^{NT}\right)}^{⊤}+\left(I-K_{k}^{NT}H_{k}^{NT}\right)P_{k|k-1}^{NT}{\left(I-K_{k}^{NT}H_{k}^{NT}\right)}^{⊤} \end{array}$$

Additionally, the EKF integrated an *h*-step-ahead state prediction stage, whose working mode is described in the next items:

1. The state corrected in (13) was used, together with the kinematic reference ${\left(x^{ref},y^{ref},\psi^{ref}\right)}_{k}^{NT}$, by the path tracking algorithm in order to calculate the dynamic reference to be followed by each wheel, ${\left({\hat{w}}_{r}^{com},{\hat{w}}_{l}^{com}\right)}_{k}^{NT}$. More details about this calculation will be given in Section 3.3.
2. From these dynamic references and the corrected rotational velocities ${\left({\hat{w}}_{r},{\hat{w}}_{l}\right)}_{k}^{NT}$, the dynamic controller was able to compute the control signal ${\hat{U}}_{k}^{NT}$ for the current sensing period. More details about this computation will be provided in Section 3.4.
3. Following an open-loop dynamics-based prediction, the non-linear model of the AV in (8) was iterated from the estimated state and the control signal in order to obtain the next state and output estimations, ${\hat{\xi }}_{k+1}^{NT}$ and ${\hat{z}}_{k+1}^{NT}$, respectively:
  (14) $$\begin{array}{c} \left\{\begin{array}{c} {\hat{\xi }}_{k+1}^{NT}=f\left({\hat{\xi }}_{k}^{NT},{\left(n_{1}\right)}_{k}^{NT},{\hat{U}}_{k}^{NT}\right)\\ {\hat{z}}_{k+1}^{NT}=h\left({\hat{\xi }}_{k+1}^{NT},{\left(n_{2}\right)}_{k+1}^{NT}\right) \end{array}\right. \end{array}$$
4. Finally, Steps 1–3 were repeated h−1 times to compute the rest of the values of the set of estimates $\left\{{\hat{\xi }}_{k+2}^{NT},\ldots ,{\hat{\xi }}_{k+h}^{NT}\right\}$.

### 3.3. Pure Pursuit Path Tracking Algorithm

From the desired kinematic reference ${\left(x^{ref},y^{ref},\psi^{ref}\right)}_{k}^{NT}$ and the state corrected by the EKF in period NT, ${\hat{\xi }}_{k}^{NT}$, the AV was able to infer its current position using a path tracking algorithm. As commented, in this work, pure pursuit was chosen (see, e.g., [25,26,27]), which generated the dynamic reference based on the rotational velocity for both wheels, i.e., ${\left({\hat{w}}_{r}^{com},{\hat{w}}_{l}^{com}\right)}_{k}^{NT}\equiv {\left({\hat{y}}_{p}^{com}\right)}_{k}^{NT}$, in order to reach properly the next point of the desired trajectory.

The reference generator was in charge of providing the pure pursuit algorithm with a sequence of *h*-step-ahead kinematic references, $\left\{{\left(x^{ref},y^{ref},\psi^{ref}\right)}_{k}^{NT},{\left(x^{ref},y^{ref},\psi^{ref}\right)}_{k+1}^{NT},\ldots ,{\left(x^{ref},y^{ref},\psi^{ref}\right)}_{k+h}^{NT}\right\}$. From these references and the *h*-step-ahead estimations, the AV path tracking was able to establish the set of *h* future dynamic references, $\left\{{\left({\hat{y}}_{p}^{com}\right)}_{k}^{NT},{\left({\hat{y}}_{p}^{com}\right)}_{k+1}^{NT},\ldots ,{\left({\hat{y}}_{p}^{com}\right)}_{k+h}^{NT}\right\}$.

The pure pursuit algorithm was based on the computation of the curvature $\bar{\gamma }$ that a vehicle must adopt from its current position (x,y) to a target position (x+Δx,y+Δy). A circle of radius *r* that joins both points can be considered, the center of the circle being in x+Δx+d. In addition, the distance to the target point is *L*.

Therefore, it is possible to express:

(15) $$\begin{array}{cc} r & =\Delta x+d\\ L^{2} & ={\left(\Delta x\right)}^{2}+{\left(\Delta y\right)}^{2} \end{array}$$

being the curvature:

(16) $$\bar{\gamma }=\frac{{\left(\Delta x\right)}^{2}+{\left(\Delta y\right)}^{2}}{2\Delta x}$$

and the so-called pure pursuit control law:

(17) $$\bar{k}=\frac{1}{\bar{\gamma }}=\frac{2\Delta x}{L^{2}}$$

Therefore, the control law $\bar{k}$ is proportional to the lateral shift and inversely proportional to the square of *L*.

The pure pursuit path tracking algorithm requires determining the target, reference position ${\left(x_{PP}^{ref},y_{PP}^{ref}\right)}_{k}^{NT}$ located at a minimum distance from the current position ${\left(\hat{x},\hat{y}\right)}_{k}^{NT}$, i.e., the so-called Look Ahead Distance (LAD), not considering the nearest points in the prescribed trajectory. This procedure avoids a severe correction, and hence, it leads to a soft movement. Assuming ${\left(x_{PP}^{ref},y_{PP}^{ref}\right)}_{k}^{NT}$ and the current position and orientation ${\left(\hat{x},\hat{y},\hat{\psi }\right)}_{k}^{NT}$, the pure pursuit control law $\bar{k}$ can be expressed as follows:

(18) $$\bar{k}=2\frac{\left(y_{PP}^{ref}-\hat{y}\right)\cos\left(\hat{\psi }\right)-\left(x_{PP}^{ref}-\hat{x}\right)\sin\left(\hat{\psi }\right)}{{\left(x_{PP}^{ref}-\hat{x}\right)}^{2}+{\left(y_{PP}^{ref}-\hat{y}\right)}^{2}}$$

For a desired linear velocity ${\left(v^{ref}\right)}_{k}^{NT}$, the rotational velocity reference ${\left(\omega^{ref}\right)}_{k}^{NT}$ can be calculated as:

(19) $${\left(\omega^{ref}\right)}_{k}^{NT}={\left(v^{ref}\right)}_{k}^{NT}\bar{k}$$

As a summary, these are the steps to be followed when the main loop of the pure pursuit algorithm is implemented:

1. Generation of the future reference for the robot, ${\left(x_{PP}^{ref},y_{PP}^{ref}\right)}_{k}^{NT}$: From the desired kinematic reference and the Look Ahead Distance (LAD), the nearest point to the future path tracking that was located far away from the LAD was calculated.
2. Control law computation: From ${\left(x_{PP}^{ref},y_{PP}^{ref}\right)}_{k}^{NT}$ and the position and orientation estimate provided by the EKF, ${\left(\hat{x},\hat{y},\hat{\psi }\right)}_{k}^{NT}$, the control law $\bar{k}$ was computed by using (18), and then, ${\left(\omega^{ref}\right)}_{k}^{NT}$ was calculated for each wheel by using (19), requiring a desired ${\left(v^{ref}\right)}_{k}^{NT}$. Finally, from these data, ${\left({\hat{w}}_{r}^{com},{\hat{w}}_{l}^{com}\right)}_{k}^{NT}\equiv {\left({\hat{y}}_{p}^{com}\right)}_{k}^{NT}$ could be calculated:
  (20) $${\left(\begin{array}{c} {\hat{w}}_{r}^{com}\\ {\hat{w}}_{l}^{com} \end{array}\right)}_{k}^{NT}=\left(\begin{array}{cc} 1/r_{r} & b/r_{r}\\ 1/r_{l} & -b/r_{l} \end{array}\right){\left(\begin{array}{c} v^{ref}\\ \omega^{ref} \end{array}\right)}_{k}^{NT}$$
  where *b* was previously defined as half of the distance between the wheels and $r_{r}$ and $r_{l}$ as the radius of each wheel.

### 3.4. Dual-Rate Controller

In this work, in order to reach the desired control performance, a dual-rate controller was used. From NT-period signals such as the dynamic reference ${\left({\hat{y}}_{p}^{com}\right)}_{k}^{NT}$ generated by the pure pursuit path tracking algorithm and the estimated, corrected rotational velocities ${\left({\hat{y}}_{p}\right)}_{k}^{NT}$ obtained by the EKF, the dynamic dual-rate controller computed *N* control actions in period *T* for each wheel inside the current sensor period NT. This control signal was previously defined as ${\hat{U}}_{k}^{NT}\;=\;{\left({\hat{u}}_{k}^{T},{\hat{u}}_{k+1}^{T},\ldots ,{\hat{u}}_{k+N-1}^{T}\right)}^{⊤}$. Following this operation mode for the next *h* dynamic references and rotational velocities, $\left\{{\left({\hat{y}}_{p}^{com}\right)}_{k+1}^{NT},\ldots ,{\left({\hat{y}}_{p}^{com}\right)}_{k+h}^{NT}\right\}$ and $\left\{{\left({\hat{y}}_{p}\right)}_{k+1}^{NT},\ldots ,{\left({\hat{y}}_{p}\right)}_{k+h}^{NT}\right\}$, respectively, the set of future control actions $\left\{{\hat{u}}_{k+N}^{T},\ldots ,{\hat{u}}_{k+hN-1}^{T}\right\}$ can be obtained.

Different alternatives can be followed to design a dual-rate controller (see, e.g., in [37,38]). In this case, the model-based dual-rate controller design described in [37] was chosen, where the cascade structure of the controller included:

- A slow-rate sub-controller: $G_{1}^{NT}\left(z^{N}\right)=u_{1,k}^{NT}/e_{k}^{NT}$.
- A digital hold: $H^{NT,T}\left(z\right)=u_{1,k}^{T}/{\left[u_{1,k}^{NT}\right]}^{T}$.
- A fast-rate sub-controller: $G_{2}^{T}\left(z\right)={\hat{U}}_{k}^{T}/u_{1,k}^{T}$.

The input of $G_{1}^{NT}\left(z^{N}\right)$ was the error signal $e_{k}^{NT}={\left({\hat{y}}_{p}^{com}\right)}_{k}^{NT}-{\left({\hat{y}}_{p}\right)}_{k}^{NT}$. Note that the output of $G_{1}^{NT}\left(z^{N}\right)$ (i.e., $u_{1,k}^{NT}$) was expanded, ${\left[u_{1,k}^{NT}\right]}^{T}$, before being injected into the digital hold $H^{NT,T}\left(z\right)$. The expand operation implied filling the slow-rate signal with zeros at the fast-rate instants (more details can be found in [37]). Then, the digital hold obtained its output $u_{1,k}^{T}$ by means of:

$$H^{NT,T}\left(z\right)=\frac{1-z^{-N}}{1-z^{-1}}$$

which in conclusion resulted in the sub-controller output $u_{1,k}^{NT}$ being repeated *N* times. From the consideration of M(s) as the desired closed-loop control performance of the original continuous-time system design, the sub-controllers $G_{1}^{NT}\left(z^{N}\right)$ and $G_{2}^{T}\left(z\right)$ were designed as follows:

(21) $$\begin{array}{ccc} G_{1}^{NT}\left(z^{N}\right) & = & \frac{1}{1-M^{NT}\left(z^{N}\right)} \end{array}$$

(22) $$\begin{array}{ccc} G_{2}^{T}\left(z\right) & = & \frac{M^{T}\left(z\right)}{G_{p}^{T}\left(z\right)} \end{array}$$

where $G_{p}^{T}\left(z\right)$ was presented in (5) and came from the discretization of the continuous-time plant model in period *T* under ZOH conditions and $M^{T}\left(z\right)$ and $M^{NT}\left(z^{N}\right)$ are the discretization of M(s) in periods *T* and NT, respectively, using ZOH techniques, as well.

## 4. Cost Indexes for Control Performance

In this section, three cost indexes closely related to control performance will be presented. By means of these indexes, the remote control solution may be compared with a conventional, single-rate control strategy. Similarly to [46], the indexes used were:

- $J_{1}$, which is based on the $\ell_{2}$-norm, and its goal is to provide a measure about how accurately the path was followed:
  (23) $$J_{1}=\sum_{k=1}^{l}\min_{1\leq k^{\prime }\leq l}\sqrt{{\left\{x_{k}^{NT}-{\bar{x}}_{k^{\prime }}^{NT}\right\}}^{2}+{\left\{y_{k}^{NT}-{\bar{y}}_{k^{\prime }}^{NT}\right\}}^{2}}$$
  where *l* is the number of iterations required by the AV to reach the final point of the path, ${\left(x,y\right)}_{k}^{NT}$ is the current AV position, and ${\left(\bar{x},\bar{y}\right)}_{k^{\prime }}^{NT}$ is the nearest kinematic position reference to the current AV position.
- $J_{2}$, which is based on the $\ell_{\infty }$-norm and is defined to know the maximum difference between the desired path and the current AV position:
  (24) $$J_{2}=\max_{1\leq k\leq l}\left\{\min_{1\leq k^{\prime }\leq l}\sqrt{{\left\{x_{k}^{NT}-{\bar{x}}_{k^{\prime }}^{NT}\right\}}^{2}+{\left\{y_{k}^{NT}-{\bar{y}}_{k^{\prime }}^{NT}\right\}}^{2}}\right\}$$
- $J_{3}$, which measures the total amount of time (in seconds) elapsed to arrive at the final destination:
  (25) $$J_{3}=lNT$$

## 5. Application

In this section, control properties reached by the remote control solution will be compared to those achieved by several single-rate, conventional control approaches defined at different rates. The section is split into two parts. Firstly, the application data will be presented, that is the different parameters for the AV model, network, and control systems. Secondly, the cost indexes introduced in Section 4 will be evaluated for the different control approaches by means of a Truetime application [42]. The comparison results will show that the proposed remote control solution was able to keep the desired control properties for the AV path tracking, despite sensing at a slow rate, the existence of process and measurement noises, and considering time-varying network-induced delays and packet disorder. In addition, the control proposal achieved a significant reduction of the network bandwidth.

### 5.1. Data

**Figure 4:**
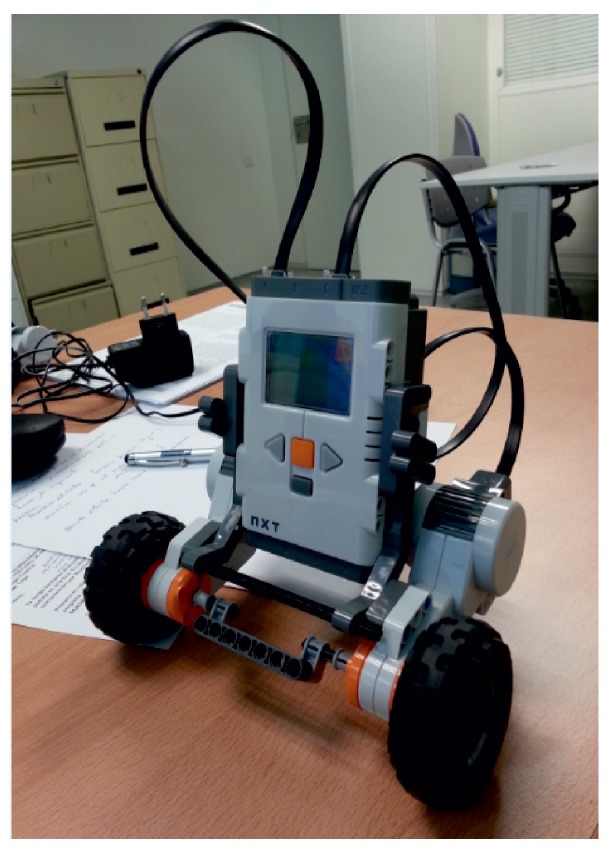
Lego robot.

These were the data used in the application:

- The AV was a Lego robot with two wheel motors (shown in Figure 4). Considering a similar model for both motors, the dynamic model for the relation between rotational velocity of the wheel and control signal is:
  (26) $$G_{p}\left(s\right)=\frac{Y_{p}\left(s\right)}{\hat{U}\left(s\right)}=\frac{0.1276}{0.1235s+1}$$
  where the output is in rad/s and the input in V. From (26), the consequent state space realization (4) can be obtained in order to be used in (7) to calculate the rotational velocities and then the rest of the elements of the state estimated by the EKF and the *h*-step-ahead state prediction stage.
- As typical in Ethernet environments [47], a generalized exponential distribution for the time-varying network-induced delays was assumed, in this case being the maximum time delay $\tau_{max}$ = 0.17 s. In order to avoid packet disorder, the sampling time NT was chosen to be NT = 0.2 s.
- From (26) and following classical procedures [48,49], a PI controller can be designed in order to achieve certain specifications. Taking into consideration this typical PI configuration:
  (27) $$u\left(t\right)=K_{p}\left[e\left(t\right)+\frac{1}{T_{i}}\int_{0}^{t}e\left(\tau \right)d\tau \right]$$
  the controller’s gains will be tuned such as $K_{p}$ = 6 and $T_{i}$ = 0.12. In order to obtain a satisfactory control performance, the actuation period was selected to be *T* = 0.1 s, and hence, the multiplicity was *N* = 2. From these values and the discretization of (26) in period *T*, a dual-rate PI controller can be designed by means of (21) and (22), bringing about:
  (28) $$\begin{array}{c} G_{1}^{NT}\left(z^{N}\right)=\frac{z^{2}-0.4734z+0.05731}{z^{2}-1.191z+0.1914} \end{array}$$
  (29) $$\begin{array}{c} G_{2}^{T}\left(z\right)=\frac{6.576z^{2}-5.78z+1.27}{z^{2}-0.9578z+0.2394} \end{array}$$
- For the comparison between dual-rate and single-rate control approaches, the continuous-time PI controller in (27) was discretized in the different periods *T* and NT. The single-rate controllers are:
  (30) $$G_{r}^{T}\left(z\right)=\frac{6z-1}{z-1}$$
  (31) $$G_{r}^{NT}\left(z^{N}\right)=\frac{6z+4}{z-1}$$
- The control solution was evaluated under different levels of noise in order to study the effect of the process and sensor noises on the performance. Let us consider a lower level of noise, where both noise signals are multiplied by a lower factor *F* = 0.1, and a higher level of noise, where *F* = 0.45. By simulation, it was checked that, from *F* = 0.45, the robustness of the control proposal may be compromised.
- Finally, the reference to be followed included a sequence of four right angles.

### 5.2. Results

In the next figures, the path reference is depicted in black, and the trajectory followed by the AV is shown in blue.

**Figure 5:**
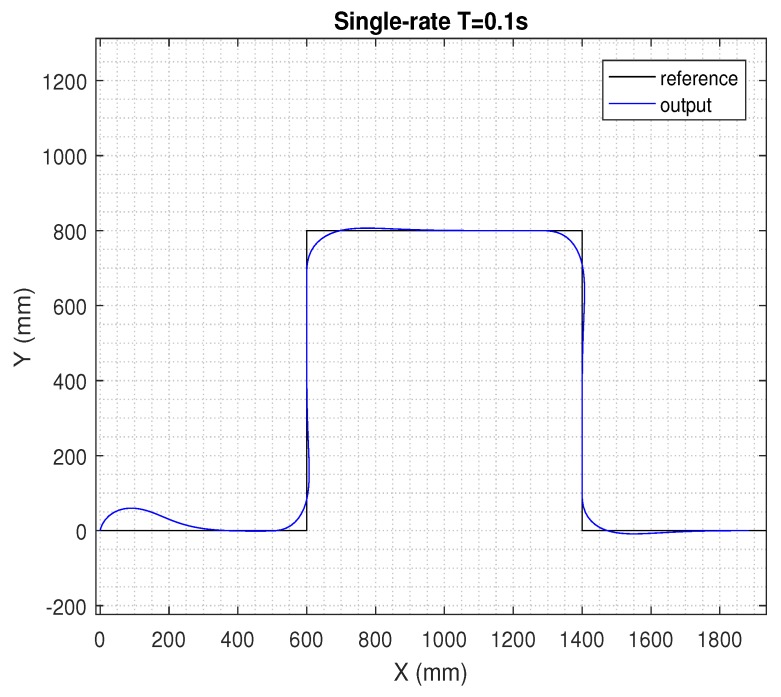
Results for the single-rate control at *T* = 0.1 s.

Figure 5 shows the results for the single-rate control in period *T* = 0.1 s in (30). In this simulation, neither delays and noise signals, nor additional control techniques (such as EKF, *h*-step-ahead prediction stage, and packet-based control) were considered. Let us define the behavior depicted in Figure 5 as the nominal behavior.

**Figure 6:**
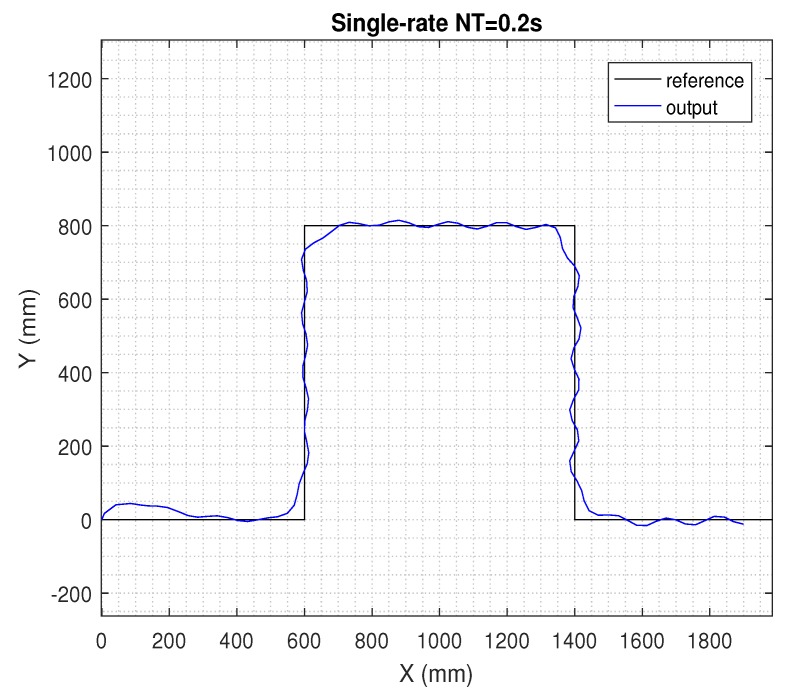
Results for the single-rate control at *NT* = 0.2 s.

In Figure 6, the sampling period was increased twice, and then, the single-rate control in period NT = 0.2 s in (31) was considered. The rest of simulation conditions were the same as in the previous case. Now, compared to the single-rate version in period *T* = 0.1 s, the control performance clearly worsened when the AV tried to track the path.

**Figure 7:**
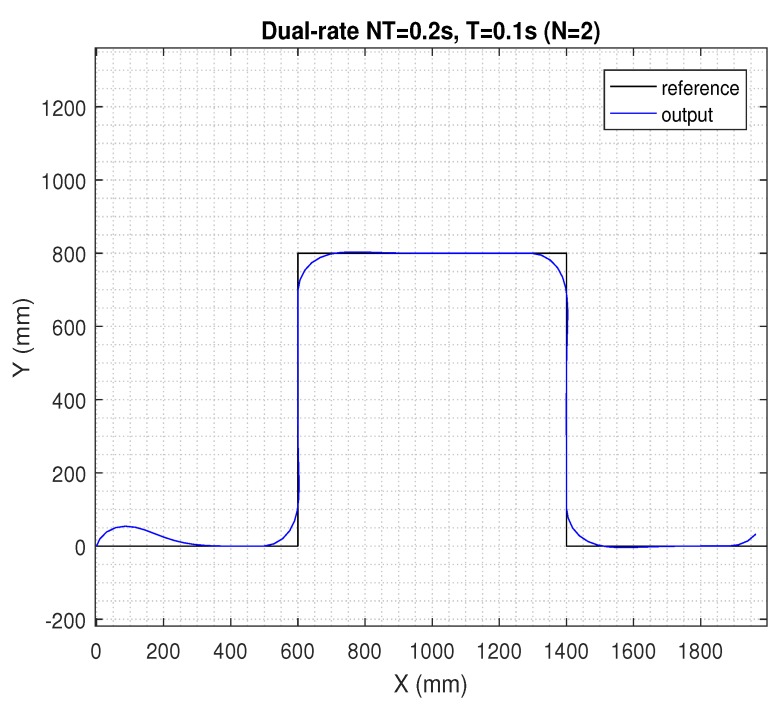
Results for the dual-rate control at *NT* = 0.2 s and *T* = 0.1 s.

**Figure 8:**
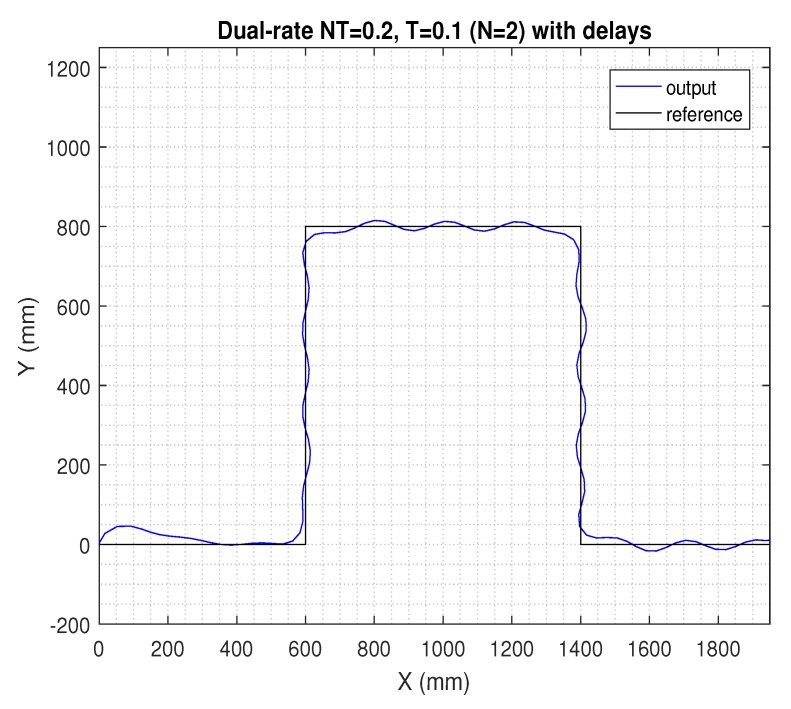
Results for the dual-rate control with delays.

If the sensing period was kept at NT = 0.2 s, but the actuation period was *N* = 2-times faster (i.e., in period *T* = 0.1 s), the dual-rate control scheme in (28) and (29) could be taken into account, which was able to maintain a satisfactory control performance, being very similar to the nominal one (see Figure 7). However, if time-varying network-induced delays were included in the dual-rate control framework, the AV’s behavior clearly worsened, as depicted in Figure 8.

**Figure 9:**
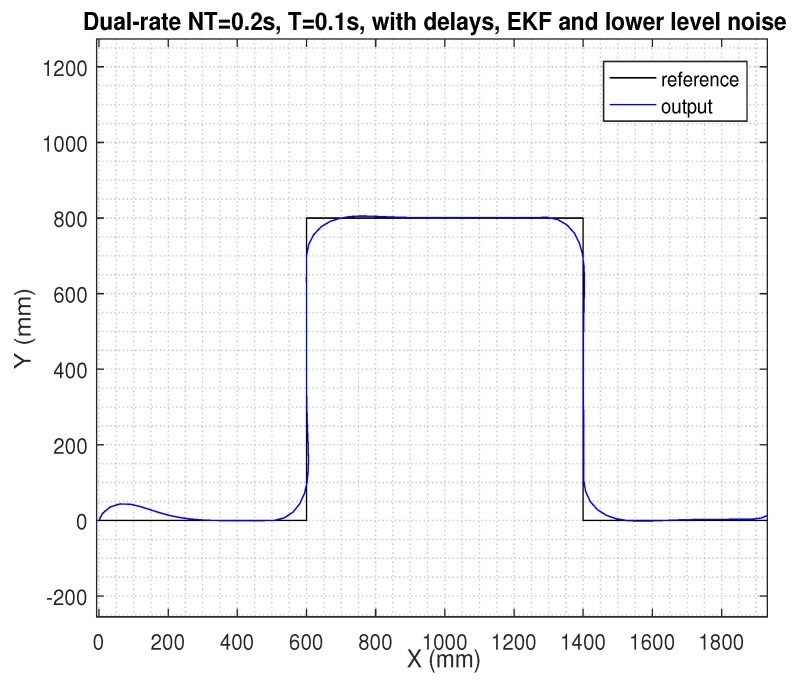
Results for the control proposal (EKF, prediction stage, and packet-based control) with delays and a lower level of noise.

**Figure 10:**
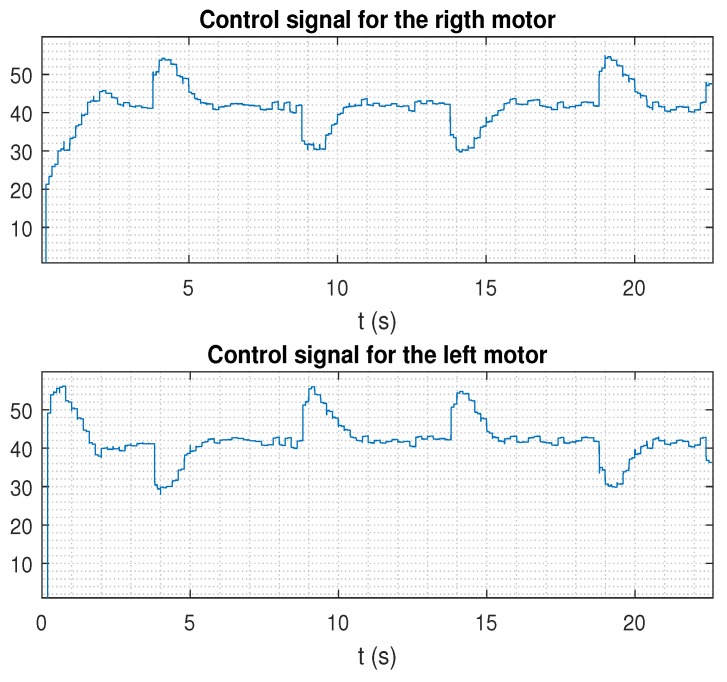
Control signal for the control proposal (EKF, prediction stage, and packet-based control) with delays and a lower level of noise.

Finally, despite existing time-varying delays and noise, if the additional control techniques proposed in this work such as EKF, *h*-step-ahead prediction stage, and packet-based control were integrated in the dual-rate control scheme, control performance was clearly improved, keeping the nominal behavior (see in Figure 9, where the case for the lower level of noise was considered; Figure 10 shows the control signal for this case). In addition, state estimates can be received at the remote server for further upper level tasks. In Table 1, an example considering *h* = 1 is given, where the set of estimations was $[({\hat{w}}_{r},{\hat{w}}_{l},\hat{x},\hat{y},{\hat{\psi )}}_{k},\left({\hat{w}}_{r},{\hat{w}}_{l},\hat{x},\hat{y},{\hat{\psi )}}_{k+1}\right]$. Note that the first value of the set of estimations was the corrected system state, and the second value was the estimation of the next state. A slight correction of the state can be observed iteration by iteration.

**Figure 11:**
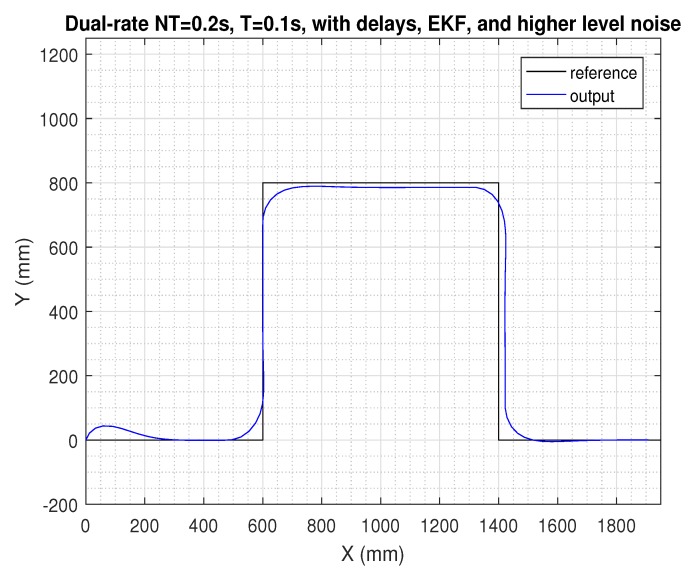
Results for the control proposal (EKF, prediction stage, and packet-based control) with delays and a higher level of noise.

**Figure 12:**
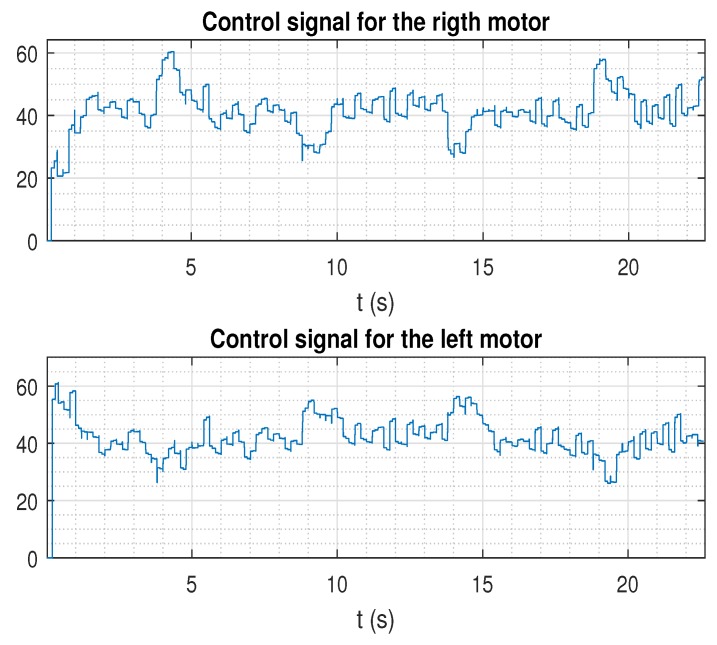
Control signal for the control proposal (EKF, prediction stage, and packet-based control) with delays and a higher level of noise.

The two last figures are presented (Figure 11 and Figure 12), where the same previous control solution was simulated, but now considering the case for the higher level of noise. As depicted, the control signal was affected by this significant magnitude of noise, which negatively affected path tracking control. Since this case represents an extreme level of noise, it will not be considered in the next study.

To analyze the previous conclusions in more detail, the cost indexes presented in Section 4 were calculated for every scenario. Table 2 shows these results, where each scenario is represented by the following letters:

- a: single-rate control scenario in period *T*.
- b: single-rate control scenario in period NT.
- c: dual-rate control scenario.
- d: dual-rate control scenario with delays.
- e: dual-rate control scenario, adding EKF, *h*-step-ahead prediction stage, and packet-based control. Delays and a lower level of noise were considered.

As previously mentioned, the desired, nominal performance was presented by Scenario a, and hence, its $J_{1},J_{2},J_{3}$ showed the reference values to carry out the comparison. As expected, Scenarios b and d showed the worst $J_{1}$ (62% worse than the nominal value), since the desired trajectory was inaccurately followed by the AV. Scenarios c and e showed a similar $J_{1}$ as Scenario a (even 1% better). Regarding index $J_{2}$, it was worsened around 16% by Scenarios b and d compared to the nominal performance, which was practically reached by Scenarios c and e. Index $J_{3}$ presented similar values for every case, differing only by ±1% with respect to the nominal value. Finally, it is worthy to note that Scenarios b, c, d, and e were able to reduce the use of the network by 50%, due to sampling it twice slower. This fact implies considerable bandwidth saving.

In summary, the proposed remote control solution was able to keep the desired control properties for the AV path tracking, despite slow sensing and the existence of noise and wireless communication problems such as time-varying delays. In addition, the control proposal significantly reduced network resource usage (50%), avoided packet disorder, and provided the remote server with future system information.

## 6. Conclusions

A novel remote control solution for AV path tracking was presented, which integrated dual-rate control, packed-based control, extended Kalman filtering, and prediction-based control techniques. A desired, nominal control performance was defined by a single-rate control at a fast rate in an ideal framework, where no wireless communication problems (such as time-varying delays and packet disorder) and no noise effect were considered. The proposal was able to reach the nominal behavior, despite communication problems and noise existing and despite sensing at a slow rate due to technical constraints introduced by the zenithal camera. In addition, the control approach enabled lessening the amount of transmission through the network, which can be decreased *N* times in comparison with the ideal framework, resulting in significant bandwidth savings.

## Figures and Tables

**Table 1:** Example for *h* = 1.

*k*	$({\hat{w}}_{r},{\hat{w}}_{l},\hat{x},\hat{y},{\hat{\psi )}}_{k}$	${\left({\hat{w}}_{r},{\hat{w}}_{l},\hat{x},\hat{y},\hat{\psi }\right)}_{k+1}$
0	$\left(\left(0.5349,0.5349,0.4621,2.9599\right)\cdot 10^{-5},-30\right)$	(2.3264,5.3668,9.5421,19.3122,−30.3040)
1	(2.3264,5.3668,9.5421,19.3122,−30.3040)	(3.1705,6.7276,29.7038,38.3284,−30.6598)
2	(3.1700,6.7265,29.6994,38.3267,−30.6597)	(3.5737,6.9281,56.5393,50.3383,−30.9951)
3	(3.5739,6.9284,56.5408,50.3384,−30.9951)	(3.8830,6.7774,86.1327,54.2475,−31.2846)

**Table 2:** Cost indexes for each scenario.

Index	a	b	c	d	e
$J_{1}$	1043.4	1671.8	1029.9	1684.4	1030.0
$J_{2}$	38.76	44.55	38.33	44.33	38.97
$J_{3}$	22.0 s	22.4 s	22.0 s	21.6 s	21.6 s

## Abbreviations

The following abbreviations are used in this manuscript:

AV	Autonomous Vehicle
IMU	Inertial Measurement Unit
EKF	Extended Kalman Filter
ZOH	Zero Order Hold
LAD	Look Ahead Distance
